# Trust in the Danger Zone: Individual Differences in Confidence in Robot Threat Assessments

**DOI:** 10.3389/fpsyg.2022.601523

**Published:** 2022-03-31

**Authors:** Jinchao Lin, April Rose Panganiban, Gerald Matthews, Katey Gibbins, Emily Ankeney, Carlie See, Rachel Bailey, Michael Long

**Affiliations:** ^1^Institute for Simulation and Training, University of Central Florida, Orlando, FL, United States; ^2^Air Force Research Laboratory, Dayton, OH, United States; ^3^Department of Psychology, George Mason University, Fairfax, VA, United States

**Keywords:** human–robot interaction, autonomous systems, trust, confidence, mental models, threat, emotion, individual differences

## Abstract

Effective human–robot teaming (HRT) increasingly requires humans to work with intelligent, autonomous machines. However, novel features of intelligent autonomous systems such as social agency and incomprehensibility may influence the human’s trust in the machine. The human operator’s mental model for machine functioning is critical for trust. People may consider an intelligent machine partner as either an advanced tool or as a human-like teammate. This article reports a study that explored the role of individual differences in the mental model in a simulated environment. Multiple dispositional factors that may influence the dominant mental model were assessed. These included the Robot Threat Assessment (RoTA), which measures the person’s propensity to apply tool and teammate models in security contexts. Participants (*N* = 118) were paired with an intelligent robot tasked with making threat assessments in an urban setting. A transparency manipulation was used to influence the dominant mental model. For half of the participants, threat assessment was described as physics-based (e.g., weapons sensed by sensors); the remainder received transparency information that described psychological cues (e.g., facial expression). We expected that the physics-based transparency messages would guide the participant toward treating the robot as an advanced machine (advanced tool mental model activation), while psychological messaging would encourage perceptions of the robot as acting like a human partner (teammate mental model). We also manipulated situational danger cues present in the simulated environment. Participants rated their trust in the robot’s decision as well as threat and anxiety, for each of 24 urban scenes. They also completed the RoTA and additional individual-difference measures. Findings showed that trust assessments reflected the degree of congruence between the robot’s decision and situational danger cues, consistent with participants acting as Bayesian decision makers. Several scales, including the RoTA, were more predictive of trust when the robot was making psychology-based decisions, implying that trust reflected individual differences in the mental model of the robot as a teammate. These findings suggest scope for designing training that uncovers and mitigates the individual’s biases toward intelligent machines.

## Introduction

Technological advancements are rapidly increasing the scope for collaboration between humans and artificial agents, including robots. Human–robot teaming (HRT) is especially important for extending operational capabilities in the military context, given that robots can endure harsh environments and prolonged periods of operation (Wynne and Lyons, 2018). Artificial intelligence (AI) also supports rapid processing of information under time pressure, in some cases far beyond human capabilities. However, teaming with artificial systems raises human factors challenges, including optimization of trust (Chen and Barnes, 2014). Antecedents of trust in conventional automation are quite well understood (Lee and See, 2004; Hoff and Bashir, 2015). However, novel features of intelligent autonomous systems such as incomprehensibility (Muggleton et al., 2018) and social agency (de Graaf and Allouch, 2013) may influence trust in ways that current models do not fully accommodate (Matthews et al., 2016, 2020). Furthermore, people vary in their perceptions of autonomous systems, leading to individual differences in trust antecedents (Schaefer et al., 2016). The present study investigated predictors of trust using a simulation of HRT in a security context and their variation with contextual factors.

The remainder of this introduction is structured as follows: First, we review evidence that various individual difference factors predict trust in robots including stable attitudes toward technology and automation (see Section “Predictors of Trust in Intelligent Security Robots”). However, individual differences in attitudes toward autonomous systems are not well understood. We review evidence that trust in an autonomous robot may reflect the person’s mental models for understanding its advanced functions, including capabilities for independent analysis and action and for teamwork (see Section “Unique Challenges of Trusting Autonomous Machines”). Individuals who see the robot as a humanlike teammate rather than a highly advanced tool may be more disposed to trust it when it exercises autonomous judgment and social agency. Several existing trust scales may capture elements of the mental model, but there is a lack of systematic research on the individual’s dominant model. Instruments that assess attitudes toward robots within teaming scenarios may provide a means for mental model assessment. The context for HRT is also important; the mental model activated in any specific teaming situation depends on the interaction between the person’s dispositions and contextual cues (see Section “Contextual Factors”). Individuals disposed to fear robots may not trust the machine, especially in threatening contexts such as security operations. Based on this analysis, we develop hypotheses for contextual and dispositional predictors of trust that could be tested in a simulation study (see Section “Present Study”).

### Predictors of Trust in Intelligent Security Robots

Technology utilizing AI for enhancing threat detection is advancing rapidly. Existing military and law enforcement applications include surveillance of vehicles and people, detecting explosives, analysis of data from the internet such as social media profiles and financial transactions, and cyber defense (Svenmarck et al., 2018; Raaijmakers, 2019). AI can be implemented through deep learning (DL) that is trained to recognize suspicious objects and people within a specific context (Svenmarck et al., 2018). Security robots with AI capabilities can guard homes and installations, rescue survivors in disaster areas, and support military Warfighters (Theodoridis and Hu, 2012). In this context, “intelligence” is defined by a perception-action cycle. The robot can both identify threats in dynamic environments utilizing multiple sources of data including sensors and make decisions following identification such as reporting to a human operator or searching for additional data (Theodoridis and Hu, 2012). AI also supports capacities for autonomy (Franklin and Graesser, 1996), defined as the ability of a system to achieve goals, while operating independently of external control (Fong et al., 2018). For example, an intelligent security robot might patrol an area without continuous human direction, choosing its path by analyzing sensor data to determine the locations to investigate (e.g., Avola et al., 2016).

Advanced security robots function not only as threat detection devices, but also as decision aids that analyze the threat and suggest options for further action (Theodoridis and Hu, 2012). A central issue for human–robot teaming is thus the human’s willingness to trust the robot’s threat analysis and recommendations. There is a large human factors literature on trust in machines (Muir, 1987; Parasuraman and Riley, 1997; Lee and See, 2004) that is relevant to human–robot interaction. Definitions of trust vary, but definition of Lee and See (2004, p. 54) is typical: “the attitude that an agent will help achieve an individual’s goals in a situation characterized by uncertainty and vulnerability.” The reviews cited identify multiple factors that shape trust, including the competence and dependability of the machine, the operator’s experiences of working with it, and social and cultural factors that may influence operator expectations. Given that few machines are perfect, the operator must calibrate trust to match the machine’s capabilities (Muir, 1987). Trust is optimized when it is based on accurate understanding of machine strengths and weaknesses, so that the operator can benefit from the machine’s capabilities without being led astray by its errors (Parasuraman and Riley, 1997; Lee and See, 2004).

Research on operator trust in robots has identified multiple antecedents of trust related to the human operator, to the robot, and to the environment (Hancock et al., 2011). Multiple factors may interact with one another to affect qualities of the operator’s engagement with robotic partners, including trust, team performance, and allocation of taskload (Chen and Barnes, 2014). Individual difference factors that might support trust calibration and hence benefit HRT include attentional control, gaming experience, and spatial ability (Chen and Barnes, 2014). Beyond such abilities and skill, differences in stable dispositional factors that shape the operator’s motivational and cognitive biases toward a synthetic partner have been neglected. Meta-analysis of Hancock et al. (2011) found that human characteristics including individual differences were only weakly related to trust, whereas robot and environment factors had stronger impacts. However, Hancock et al. (2011) cautioned that only a limited number of studies of human characteristics were available. So, there remains uncertainty over the role of human characteristics in trust. As Hancock et al. (2011, p. 523) state, there is “a strong need for future experimental efforts on human-related, as well as environment related factors.” More specifically, research on aspects of the teaming environment such as team collaboration (i.e., shared mental models, communication, culture, and in-group membership) and tasking factors (i.e., task type, task complexity, multi-tasking requirement, and physical environment) may have neglected the role of individual differences in trust. The current study aimed to answer call of Hancock et al. (2011) by examining relevant dispositional factors that may predict individual differences in trust in HRT.

Personality factors predict trust and automation-dependence in a variety of other contexts for automation (Matthews et al., 2020), including autonomous vehicles (Choi and Ji, 2015), software (Ryan et al., 2018), and unmanned aerial systems (Lin et al., 2020). A further meta-analysis (Schaefer et al., 2016) confirmed that human characteristics were moderately related to trust in automated systems, excluding robots. Some studies of robots published following the Hancock et al. (2011) meta-analysis have demonstrated personality–trust associations. For example, Haring et al. (2013) found that the extraversion trait was positively correlated with trust in robots. Salem et al. (2015) failed to replicate this association, but they found that neuroticism was associated with lower likability of robots. Dispositional attitudes toward technology (e.g., Hegner et al., 2019) may be more predictive of trust in robots than are broad personality dimensions. Lyons and Guznov (2018) found that trust was reliably correlated with having high expectations of automation, measured using the Perfect Automation Schema (PAS) scale (Merritt et al., 2015). Here, we focused on such factors rather than general personality traits.

### Unique Challenges of Trusting Autonomous Machines

Human engagement with intelligent machines depends critically on perceptions that the machine is trustworthy (Glikson and Woolley, 2020; Lockey et al., 2021). Influences on trust may differ somewhat in autonomous systems compared to conventional automation (Schaefer et al., 2016). Systems with sufficient intelligence to operate autonomously are difficult for the human operator to understand and anticipate, implying that tolerating ambiguity and complexity may be important for trust (Matthews et al., 2016). In addition, some systems exert social agency, i.e., they can evaluate the status of the human operator and respond with teaming behaviors such as taking on additional tasks, signaling their goals and intended actions, and communicating their willingness to support the human (Wang and Lewis, 2007; Chen and Barnes, 2014; Volante et al., 2019). Wynne and Lyons (2018) highlight the need for further research on humans’ perceptions of intelligent robots. In this section, we argue that (1) mental models for robot function influence trust; (2) attributes of the robot influence the mental model; and (3) individuals have stable preferences for the mental model they apply to the robot.

#### Mental Models for Advanced Robots Influence Trust

Advanced robots and other types of intelligent agent may be viewed as either highly sophisticated tools or as teammates with some human-like qualities (Chen and Barnes, 2014; Matthews et al., 2019). Such perceptions reflect the differing mental models that operators apply to understanding their interactions with the robot (Ososky et al., 2013). Mental models may encode both explicit and implicit preferences (Merritt et al., 2013). Operator mental models of technology are not always fully accurate or complete, but they serve to direct attention and guide the user’s actions (Phillips et al., 2011). Understanding the user’s mental model may be important for designing transparency information to be compatible with the dominant model (Lyons and Havig, 2014).

Trust in autonomous systems may vary according to whether the dominant mental model defines the system as an advanced tool or teammate (Ososky et al., 2012; Perelman et al., 2017; Matthews et al., 2019). Congruence between the operator’s mental model and robot functioning enhances human understanding of the robot’s intent and hence effective teaming (Ososky et al., 2014). The appropriateness of the operator’s mental model may be especially important when the decision space is less constrained so that many ways exist for even human partners to arrive at a solution (Perelman et al., 2020).

Generally, people are more likely to trust intelligent robots to make functional analyses such as optimizing workflow in an industrial setting than to make social judgments (Glikson and Woolley, 2020). Such findings may reflect the tendency to apply a default tool model to machines. When the robot has autonomous capabilities for teamwork, perceiving it as human-like may functionally adaptive. Such perceptions support shared mental models of team roles between the human and robot, leading to improved situation assessment, situation awareness, and performance (Schuster et al., 2011). Shared mental models allow the operator to predict and react to the robot’s behavior and motivate the operator to team effectively with the system (Schuster et al., 2011). However, much of the research on factors that drive trust in advanced robots does not directly address trust calibration (Lee and See, 2004). For example, trust in a human-like robot may be misplaced if the robot does not in fact execute cognitive tasks accurately.

#### Attributes of the Robot and the Mental Model

The challenge for trust research is that the mental model active in any given interaction may vary across time, situations or tasks, and individuals (Phillips et al., 2011), so that the drivers of trust also vary. Mental model activation may depend on both robot factors such as its appearance and its task activities (Glikson and Woolley, 2020) and individual differences in personality that affect attitudes toward robots (Wynne and Lyons, 2018).

The ways in which the robot interacts with the human may bias which mental model is activated (Ososky et al., 2014). For example, consider systems that suggest music based on user preference. Kulesza et al. (2012) described a web-based interface that recommended tracks based on the user’s expressed preferences for artists and styles of music. User mental models reflected their beliefs about the application’s rules for choosing music, i.e., the models were mechanistic in nature. By contrast, virtual personal assistants (VPAs) such as Alexa and Siri express human-like personality traits that influence the user’s mental models accordingly and their engagement with the VPA’s recommendations (Poushneh, 2021; Tenhundfeld et al., 2021).

Factors such as a human-like appearance, expression of emotion, social agency and intelligence, benevolence of intent, communication abilities, and enjoyment of interaction promote acceptance of social robots (de Graaf and Allouch, 2013; Kuhnert et al., 2017; Wynne and Lyons, 2018). Review of social human–robot interactions of Lewis et al. (2018) cites additional human-like qualities that elevate trust such as physical presence, matched speech, and empathetic language and physical expression. Anthropomorphic design features such as simulated personality and a naturalistic communication style also tend to increase trust in systems that utilize AI such as robot assistants (Kiesler and Goetz, 2002) and virtual assistants (Lockey et al., 2021). However, the advent of more human-like robots does not imply that humans will always trust social robots; attributes such as unreliability (Lewis et al., 2018) or undesired personality features (Paetzel-Prüsmann et al., 2021) will tend to diminish trust. There may also be specific contexts such as healthcare in which more anthropomorphic robots are trusted less (Chui et al., 2019). The mental model might encode the robot as human-like but incompetent.

#### Individual Differences in the Mental Model

Individual differences in trust in robots are quite prevalent and appear to reflect both attitudes toward the reliability of automation (Pop et al., 2015) and toward humanoid robots (Nomura et al., 2008). People also differ in their tendencies to anthropomorphize inanimate objects (Waytz et al., 2010). However, research is lacking on measures of individual differences in mental model dominance relevant to the security context. To address this research gap, Matthews et al. (2019) conducted a study of trust that aimed to identify situational and dispositional factors relevant to threat detection in military and police settings. A new instrument, the Robot Threat Assessment (RoTA) scale, was developed using Situation Judgment Test (SJT) methodology (Lievens et al., 2008). The aim was to evaluate trust in robots performing threat evaluations in a series of scenarios, described in short text passages. Scenarios differed in terms of whether the robot performed a purely physics-based analysis such as detecting the presence of chemicals or radiation, or whether it analyzed sensor data psychologically, e.g., for fear or aggressive intent in a human target. Physics-based analyses implied the robot was reading a value from a meter or performing a simple pattern-recognition task such as matching a fingerprint to a database. Psychological analyses additionally required the robot to make an inference about distinctly human qualities including emotion, motivation, deception, and attitudes. Factor analysis of participant ratings distinguished two correlated dimensions corresponding to physics-based and psychological judgments, respectively (RoTA-Phys and RoTA-Psych), consistent with trust reflecting distinct mental models.

Matthews et al. (2019) assessed dispositional factors corresponding to operator beliefs and attitudes toward robots encoded in the “advanced tool” and “teammate” mental models. In the former case, trust may reflect individual differences in beliefs about whether the system is an effective and reliable tool. The PAS (Merritt et al., 2015) measures high expectations for automation as well as all-or-none beliefs. The PAS correlated positively with trust in both physics-based and psychological contexts, suggesting that the tool mental model tended to predominate. When the teammate mental model is active, trust may also depend on whether the system is believed to be a supportive teammate, i.e., whether it effectively performs teamwork as well as taskwork behaviors (cf., Driskell et al., 2018). Study of Matthews et al. (2019) also included the Negative Attitudes to Robots scale (NARS: Nomura et al., 2006), which assesses a range of negative reactions. Validation studies showed that scores correlated negatively with acceptance of social robots (Nomura et al., 2008), avoidant social behaviors during interactions with a robot (Ivaldi et al., 2017), and trust in robots (Schaefer, 2013). In study of Matthews et al. (2019), the NARS predicted lower trust in robots making psychological judgments, over and above the PAS. Thus, the NARS may reflect the teammate mental model, with high scores suggesting negative views of robots as teammates, and low scores indexing positive attitudes.

### Contextual Factors

Detection of threat by a human–robot team can take place in a variety of environments, varying in the objective probability of threat. For example, police officers are required to patrol city areas varying in crime rates. Contextual attributes of the environment provide cues to threat probability; high crime-rate areas are likely to have graffiti, broken windows, and damaged vehicles present. From a Bayesian perspective, these contextual cues help the officer estimate the base rates for threats, improving decision making. Depending on the base rate, the validity of a robot partner’s threat estimation might be higher or lower than the accuracy of the robot’s determinations. While people, even domain experts, are notoriously fallible as Bayesian decision makers (Heuer, 1999), it is likely that law enforcement officers factor in base rates to some degree. For example, neighborhood characteristics influence officers’ enforcement decisions (Worrall et al., 2018). Similarly, soldiers with combat experience scan for danger cues such as people with hostile facial expressions, argumentative or aggressive postures, or unusual movement patterns (Zimmerman et al., 2014). Thus, effective HRT requires the human to evaluate robot decisions within the context of base-rate likelihood of danger. Mental models that promote high trust may lead to under-weighting of contextual information and over-weighting of the robot’s recommendation. Conversely, mental models encoding negative attitudes to the robot may promote over-sensitivity to context.

Dangerous environments introduce an additional element into decision making, emotions such as fear and anxiety. The research literature demonstrates a range of biases associated with anxious emotion (Blanchette and Richards, 2010), such as increasing salience of threats (Matthews et al., 2011). Neuroticism, a trait linked to anxiety, is associated with both lower interpersonal trust and trust in automation (Szalma and Taylor, 2011), although its role in reactions to automation is under-researched (Hoff and Bashir, 2015). Traits linked to mental models for robots may also influence the relative weighing of contextual and robot-generated information, congruent with whether the attitude is positive or negative. Traits also capture typical affective reactions, and emotions may influence trust over and above cognition and attitudes (Lee and See, 2004). For example, a high scorer on the NARS might experience a visceral dislike of a robot partner. Such reactions may be more prevalent when the environmental context activates negative emotions.

### Present Study

The present study aimed to investigate whether dispositional predictors of trust in an autonomous robot were moderated by context as suggested by the mental model perspective previously introduced. It aimed to extend the Matthews et al. (2019) findings by placing the participant in an immersive virtual environment, rather than using text-based scenarios. Participants were teamed with a robot in a simulation of threat detection and analysis in an urban environment, rendered with moving 3D graphics. The task resembles that of a pilot vehicle that precedes a convoy to provide a threat assessment as part of a Close Protection Operation (Schneider, 2009). Close Protection Operators anticipate and plan for potential threats in part by examining locations along a convoy route where an attack or ambush might take place. In the current study, the participant worked with a robot partner to visually assess the level of threat present along the path of a following motorcade. The robot used various sensors to determine whether threat was present at various stops along the route. It analyzed for threats including physical threats, such as fire or radiation, and human threats, such as impending violence between citizens. The simulation included a series of scenes that differed in the level of visible danger cues, such as broken windows, to provide a manipulation of environmental context.

At each scene, the robot provided a text report on whether it judged a threat to be present or not. Similar to Matthews et al. (2019), we varied whether the robot’s evaluations were physics- or psychology-based. The manipulation involved presenting text-based transparency information (Lyons, 2013) that described either the physics- or psychology-based analyses supporting the robot’s threat evaluation. In the physics-based condition, transparency messages referred to direct inferences of threat from information such as the presence of chemicals, a metal object, or a weapon-shaped X-ray image. In the psychological condition, messages referred to inferring human emotions and intentions from cues such as elevated breathing, content of language, and body posture. Consistent with previous studies of transparency, mental models, and trust (Ososky et al., 2014), we assumed that physics-based messages would tend to activate a “tool” mental model, whereas psychological messages would prime a “human-like” model. The study manipulated type of analysis between subjects so that the robot consistently performed one type of analysis only.

We distinguished stable dispositional factors likely to bias mental model activation from situational constructs that capture the operator’s immediate attitudes toward the robot. Dispositional factors were assessed with the PAS and NARS. The study also included Human Interaction and Trust (HIT) scale of Lyons and Guznov (2018) that assesses intentions to rely on a robot. Dispositional factors were conceptualized as attributes of the individual’s stable, underlying mental model for advanced robots that guided but did not fully determine the mental model activated during the task. The situational trust response was assessed with ratings provided after each evaluation made by the robot. An overall post-task trust scale for HRI (Schaefer, 2016) was also administered as a check on the validity of the situational ratings, i.e., that ratings converged with a validated situational trust measure. Ratings of whether the robot was making psychological judgments in each scenario were also obtained. These ratings reflect the dominant mental model activated in the scenario, presumed to reflect both dispositional factors and the impact of the transparency messages.

### Research Issues and Hypotheses

The study manipulated robot analysis type and level of contextual danger cues within subjects, and robot analysis type between-subjects (to activate a consistent mental model across scenarios). The principal dependent variable in the study was self-reported trust in the robot’s decision. Other outcomes included situational ratings of threat, anxiety, and perceptions that the robot was making psychological judgments. We investigated the following research issues.

#### Robot Analysis Type and Trust

Matthews et al. (2019) found that trust in robots was higher for physics-based than for psychologically based judgments, on the basis of responses to text scenarios. We expected that any inherent bias against human-like robots would persist in an immersive scenario with richer contextual information available to the participant than text provides. We hypothesized that there would be a main effect of robot analysis type on trust (H1).

#### Contextual Danger and Trust

In many security contexts, the operator’s task requires integrating their own perceptions of danger with the additional information provided by a robot. Conflict between the human’s perceptions and the robot evaluation may call the robot’s ability into question (Perelman et al., 2020), damaging trust according to Mayer et al. (1995) ability, benevolence, and integrity model of trust. Trust was thus expected to be higher when the robot confirmed the operator’s perceptions, and lower when there is a mismatch. We manipulated danger perceptions by varying presence of cues such as broken windows and suspicious objects. We hypothesized that trust would reflect the degree of congruence between the robot decision and the visible environmental context. Trust should be highest when the robot responds “safe” in a low-danger context and “threat” in a high-danger context (H2). That is, we anticipated an interactive effect of level of danger cues and the robot’s decision. We tested whether the interaction between robot decision and context was moderated by the decision type on an exploratory basis.

#### Individual Differences in Trust: Moderator Effects of Mode of Robot Analysis

Potential correlates of situational trust were divided into two classes. The PAS, HIT, and RoTA-Phys scales evaluate trust in machine functions without reference to any human-like judgments or behaviors. Assuming that the default mental model for automation (PAS) and robots (HIT, RoTA-Phys) refers to analyses of the physical world, we hypothesized that these scales would primarily predict trust in the robot when making physics-based decisions (H3a). Conversely, the NARS and RoTA-Psych scales explicitly refer to human-like functions. Low NARS and high RoTA-Psych scores were expected to predict trust in the robot when making psychology-based decisions (H3b). The RoTA-Psych asks respondents to rate both the extent to which the robot is making a psychological judgment, and their confidence in the robot’s analysis. It was anticipated that these perceptions of robot functioning would be more predictive of trust in the psychological compared to the physics-based analysis condition.

For each scenario, participants rated their perceptions of the robot as making psychological judgments, as well as threat and anxiety. Similar to H3b, we hypothesized that perceptions of the robot as operating psychologically would correlate more strongly with trust in the psychological compared to the physics-based condition (H3c). We ran regression analyses that included mode of robot analysis as a moderator factor to test hypotheses H3a through H3c. We also investigated scenario ratings of threat and anxiety as correlates of trust, on an exploratory basis.

#### The Role of Emotion in Trust

We expected that scales for attitudes to human-like robots would be associated with perceived threat and anxiety, leading to congruent changes in trust, especially in the most threatening context. Hypotheses were tested in the psychological condition only. High NARS and low RoTA-Psych scores should correlate with threat and anxiety (H4a), and correlations between these scales and trust should approach zero with threat and anxiety controlled (H4b).

## Materials and Methods

### Participants

Sixty-two men and 56 women were recruited from the psychology student pool (age range 18–40) at a large state university in the southeastern United States. They received course credit for participation.

Previous studies (e.g., Merritt et al., 2015; Lyons and Guznov, 2018; Lyons et al., 2020) have shown large variation in the magnitudes of correlations between dispositional and situational trust measures, which made an *a priori* power analysis problematic. We aimed to run approximately 120 participants based on typical sample sizes in comparable simulation-based studies (e.g., Schaefer, 2016; Lyons and Guznov, 2018). With an *N* of 118, and for correlations of 0.3, 0.4, and 0.5, power attained was 0.88, 0.99, and 0.99, respectively (*p* < 0.05, two-tailed). Within each group of 59 participants, the equivalent power values were 0.65, 0.89, and 0.99.

### Experimental Design

The study utilized a 2 × 2 × 3 design. Robot analysis mode (physics-based, psychological) was manipulated between-subjects. Thirty-one men and 28 women were allocated at random to each of the two conditions. Participants in the two groups were similar in demographic characteristics including education level, self-reported health, and hours of sleep. Robot decision (safe, threat) and danger cue level (low, medium, and high) were manipulated within subjects.

### Simulation Environment

The simulation was designed using UnReal Game Engine4. It supported threat evaluations performed as part of a mobile protection plan, i.e., providing threat intelligence required for the close protection of dignitaries traveling in a convoy. The participant played the role of a soldier traveling the convoy route along with a robot partner with the aim of identifying possible terrorist threats. The participant had a “first-person shooter” 3-D view of traveling through an urban environment, stopping at a series of locations along the route to determine whether threatening activity was present. Participants were told that the robotic partner was equipped with multiple sensors, which it used to make its own determination of each location’s potential for threat. The participant could not see the robot directly but could view text information provided by the robot pertaining to its threat evaluations.

Specifically, the initial orientation included the following instructions: “You will play the role of an operator using a robot to investigate a potentially threatening region in an urban town. The robot uses sensors and programmed artificial intelligence to analyze the situation. It will deliver to you a threat assessment and a recommendation for action. You should evaluate the situation onscreen, then rate your confidence in the robot’s threat assessment and recommendation for action. You should make these ratings independently. For example, you might be confident in the robot’s evaluation of threat, but not its action suggestion, or vice versa. The scenarios cover a range of robot technologies that already exist or are currently being developed. You may not have heard of all of them, and we do not expect you to be familiar with the technologies. We would like you to respond by considering the general plausibility of the technology working as intended, drawing on whatever relevant knowledge you have.”

Danger cues present in the scene were manipulated to be low, medium, or high based on the number of suspicious objects, characteristics of individuals, and the condition of the buildings in the area. Objects were planted in scenes to elicit the perception of common security threats, such as an unattended bag potentially containing an improvised explosive device or a suspicious unmarked van. Individuals were either static or cycled through a series of actions, such as gesturing. There was no other movement in the scene. There were about eight individuals per scene, across the foreground and background. Individuals were manipulated to appear threatening by using large, animated gestures mixed with angry facial expressions. Buildings in the scene varied from newly built and maintained (low threat) to vacant and in disrepair (high threat). Combinations of object, building, and individual qualities were planned by the laboratory’s research associates; threat levels were confirmed by other team members uninvolved in the project. The scenarios were therefore designed as follows. In low danger scenes, buildings were new, one suspicious object was planted in the scene, and one out of three individuals seemed angry. In medium danger scenes, buildings were slightly rundown and painted in the modeling software to appear dirty but without disrepair (no boarded or broken windows). There were also two suspicious objects such as a discarded duffle bag or package and one or two individuals appeared upset and making angry or violent gestures. In the high danger scenes, the buildings appeared in disrepair with broken or boarded windows and entrances. Suspicious objects were higher in number. These included fire or smoke in the scene, and old packages or containers. Individuals in these scenes appeared ready to fight or riot, e.g., through exhibiting angry gesturing motions. Agents were carefully modeled to remove any social biases, which might influence danger perceptions. There was an even representation across scenes of gender, of light, medium, and dark complexion agents, and of younger and older individuals (appearing below or above 40 years of age).

At each scene, after approximately 20 s, the robot provided its evaluation as a text message overlaid on a portion of the screen. The message include justification for the evaluation (transparency) and a statement that the area was either safe or threatening. Table 1 shows the number of robot decisions of each type at each level of danger cues. The robot described the majority of low-danger scenes as safe and the majority of high-danger scenes as threatening, in order to maintain ecological validity. This scheme introduces a correlation between robot decision and level of danger cues. We felt that the alternative approach of having equal numbers of each robot decision type at each danger level would rapidly undermine trust in the robot and threaten the generalizability of results.

**Table 1:** Number of scenarios at each level of danger cues and robot decision.

	Low danger	Medium danger	High danger
Robot decision: safe	7	4	1
Robot decision: threat	1	4	7

**Figure 1:**
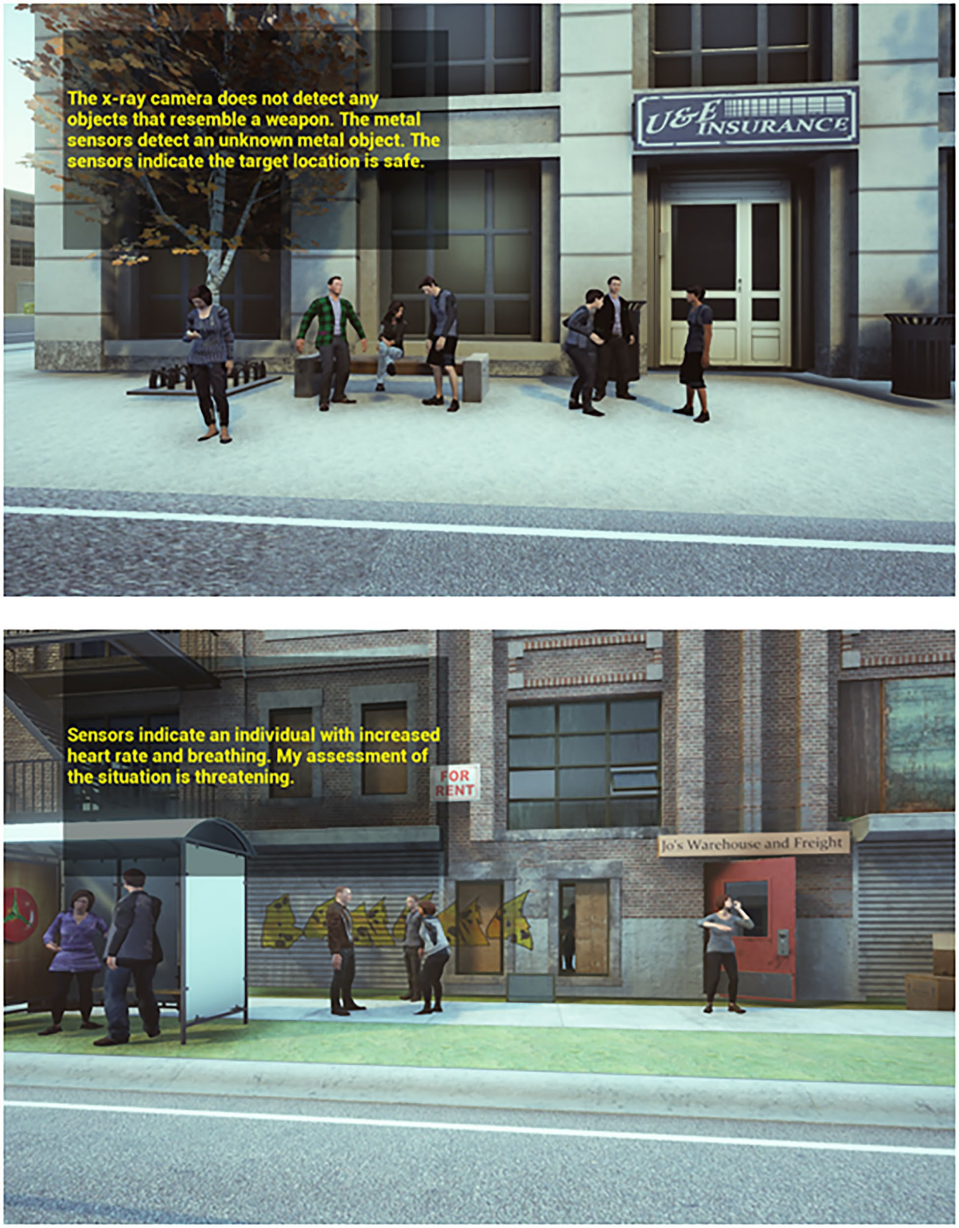
Screenshots from two scenarios. Upper screenshot has low danger-cue level, and the robot makes a physics-based evaluation of safety. Lower screenshot has high danger-cue level, and the robot makes a psychological evaluation of threat.

The mode of analysis described in the message was manipulated as follows. In the physics-based condition, the robot described making its judgment of the scene using physical or chemical cues in the environment, such as a potential fire threat determined from thermal readings. In the psychological condition, the robot partner’s threat determination was based on psychological analysis of sensor readings, such as using thermal cameras to measure facial temperature and infer suspicious stress response in individuals in the scene area. Participants were able to view the scene again up to three times, without the robot message overlaid, before proceeding to response. Figure 1 illustrates scenes differing in level of danger cues and in analysis mode.

After viewing the scene and the robot evaluation, a series of questions were overlaid on the screen, together with five-point Likert scales for response. Participants rated the level of threat, their emotional state, and the robot’s assessment of the scenario. Following response, the participant viewed the journey to the next scene. No feedback on the robot’s accuracy of threat analysis was provided.

### Measures

#### Demographics Questionnaire

A 14-item demographics questionnaire covered a range of biographical information, including gender, health state, education level, and military service experience.

#### Dispositional Attitudes Toward Robots

The NARS, PAS, HIT, and RoTA scales were used to measure people’s attitudes toward robots. The NARS (Nomura et al., 2006) consists of 14 items in three subscales to profile general attitudes toward robots: NARS_1. Negative attitude toward interaction with robots; NARS_2. Negative attitude toward social influence of robots; NARS_3. Negative attitude toward emotional interactions with robots. The NARS_3 items refer to comfort with interactions, but they were reverse-scored so that high scores refer to discomfort. Nomura et al. (2006) reported alpha coefficients for internal consistency of 0.77, 0.78, and 0.65, for the three subscales, respectively.

The PAS (Merritt et al., 2015) consists of eight items relating to people’s trust in automated systems, with two subscales: PAS_1. High expectations; PAS_2. All-or-none belief. Each item is scored on a five-point Likert scale from “strongly disagree” to “strongly agree.” Matthews et al. (2019) found that both subscales had good internal consistency (alpha = 0.82, for both).

The HIT (Lyons and Guznov, 2018) comprises 10 items asking about intentions to trust a robot partner, based on the based on the trust model of Mayer et al. (1995). Items are answered on a seven-point scale, anchored by “not at all true” and “very true.” Lyons and Guznov (2018) reported an alpha coefficient of 0.83.

The RoTA scale (Matthews et al., 2019) includes text descriptions of 20 military and security scenarios in which a human operator teams with a robot to investigate a potentially threatening event or person in a remote location. To determine threat, the robot uses physics-based (10 scenarios) or psychological analysis of sensor data (10 scenarios). Two examples of physics-based scenarios included the robot detecting traces of explosive chemicals and using a thermal camera to locate humans inside a building from heat patterns. Psychologically based scenarios included detecting aggression in facial expressions and identifying acoustic qualities of speech indicating anxiety. After each scenario, participants were asked to make three ratings on an eight-point scale: Q1. *To what extent is the robot making a psychological judgment*; Q2. *How confident are you that the robot’s analysis of the situation is correct*; Q3. *How likely are you to base your actions on the robot’s recommendations*? Alpha coefficients for 10-item scales corresponding to each question, calculated separately for physics-based and psychological scenarios, ranged from 0.81 to 0.97.

#### Scenario Ratings

Following each scenario, participants rated the likelihood of threat (0%: Secure—100%: Threatening), and their levels of five emotions, including anxiety. A further screen requested ratings of the extent to which the robot’s assessment was psychological in nature (1: Not at all—5: Very much), their confidence that their partner’s assessment was correct (1: Not at all confident—5: Very confident), and the likelihood of their acting on the robot’s recommendations (1: Not at all likely—5: Very likely). These ratings were averaged across scenarios to give overall measures of scenario perceptions, including trust, the principal dependent measure.

#### Situational Trust (Post-task)

Following task performance, participants completed a 14-item subscale measuring trust in functional capabilities of the robot, from Trust Perception Scale-HRI (TPS-HRI) of Schaefer (2016). The scale was scored as a post-task measure per instruction of Schaefer (2016). Participants rated the percentage of time that the robot displayed various attributes of trustworthiness, such as being part of the team and responsible, on an 11-point scale ranging from 0 to 100%. The TPS-HRI was included primarily to check the validity of the situational trust ratings; the 14-item subscale was used rather than the 40-item full scale due to time constraints.

### Procedure

**Figure 2:**
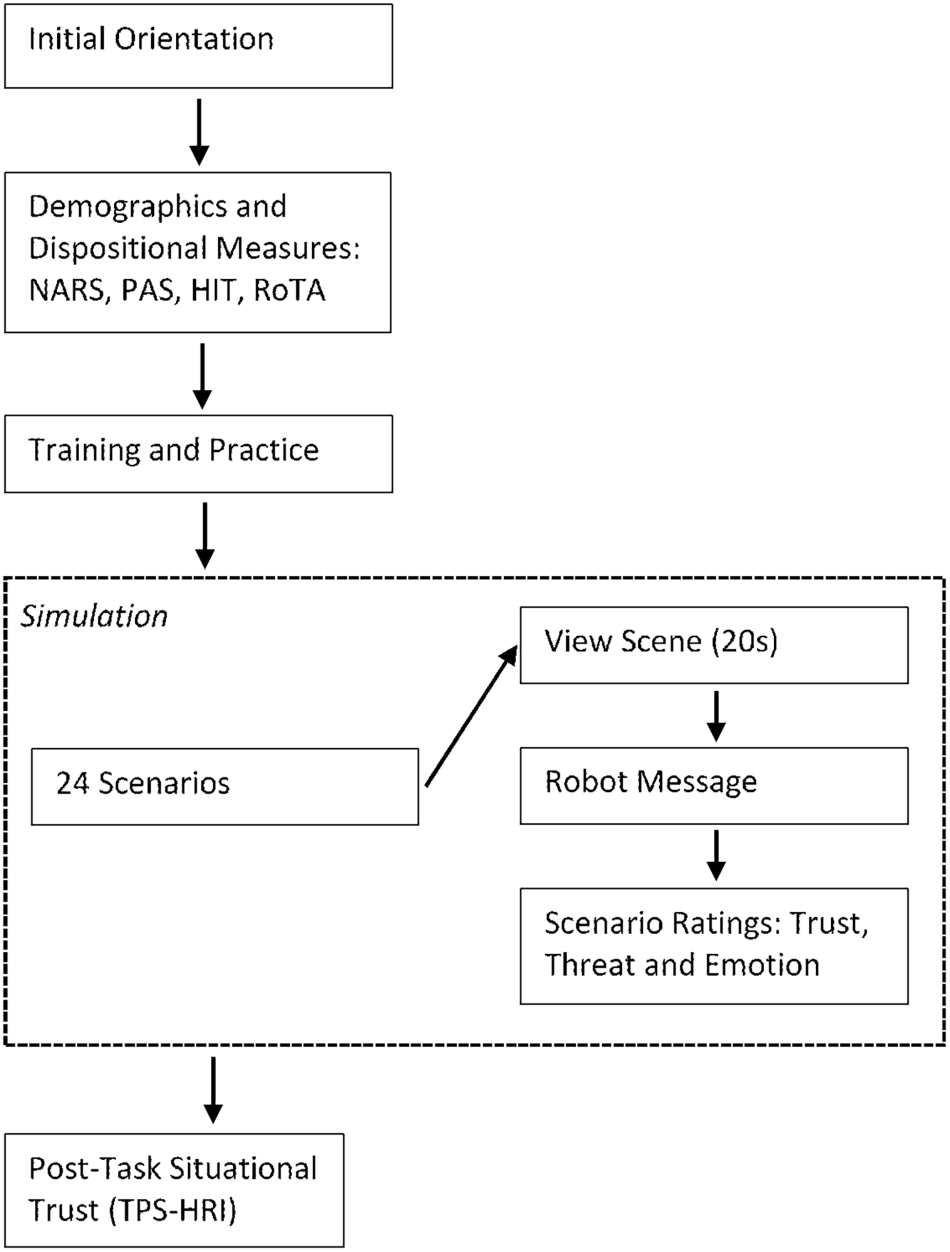
Summary of the procedure.

Figure 2 summarizes the procedure, comprising the following steps.

#### Initial Orientation

Participants were given a general description of the study and the simulation and provided informed consent. The participants were allocated at random to one of the two robot analysis mode conditions, either physical or psychological.

#### Demographics and Dispositional Measures

Participants completed a questionnaire packet including demographic questions and the NARS, PAS, HIT, and RoTA scales.

#### Training and Practice

Participants viewed a training PowerPoint slide deck that described the aims of the security mission and the role of the robot, illustrated the simulation environment, and provided instructions for rating their evaluations of the robot’s decisions. Instructions described either the robot’s use of physical and chemical sensor data, or its capacity to analyze psychological characteristics according to the experimental condition to which the participant was allocated. They were instructed that “Although you have the robot to assist you, you still need to examine each situation carefully to look for signs of terrorist activities, such as weapons, suspicious objects, aggressive behavior, etc.” Participants then completed a hands-on training session, guided by the researcher, which walked them through a threat evaluation scenario, provided practice in rating threat, emotion, and the robot’s decisions, and verified that they fully understood the task and the rating procedure.

#### Simulation Task

Participants responded to a sequence of 24 scenarios, as specified in Table 1. Scenarios were the same in the two robot analysis mode conditions, but the transparency messages from the robot differed. Danger cue level and robot decision were varied in a pseudo-random order. For each scenario, participants viewed the scene for 20 s, received the robot transparency message, and completed a set of ratings overlaid on the screen. Ratings included the extent to which the robot was making a psychological judgment and two trust evaluations: confidence in the robot’s judgment and willingness to accept an action recommendation from the robot. They also included perceived threat and levels of five emotions including anxiety.

#### Post-task Situational Trust

Finally, participants completed a further questionnaire packet including the TPS-HRI, prior to debriefing.

## Results

### Overview

Following manipulation checks, an ANOVA was run to investigate effects of the experimental manipulations on trust ratings, testing H1 and H2. This ANOVA suggested that effects were similar for the two ratings utilized, focusing on confidence in the robot and willingness to act on its recommendations, respectively. For correlational analyses, these ratings were averaged to provide an overall situational trust measure. Following a check on predictor intercorrelations, we report correlations of the averaged trust measure with (1) dispositional measures and (2) scenario ratings. Correlations were compared across the two robot analysis modes, testing H3a, H3b, and H3c. Multiple regressions were run to provide a formal test of the moderator effects of robot analysis condition on predictors of trust.

The final set of analyses addressed associations between dispositional measures and emotional response, and the extent to which dispositional measures remained correlated with trust when emotional response was controlled. The preceding analyses showed that the dispositional trust measures were only weakly correlated with trust in the physics-based analysis condition. Thus, the role of emotion was of little relevance in this condition. By contrast, multiple dispositional measures were associated with trust in the psychological condition, so that emotional factors might potentially be more relevant to understanding individual differences in trust. Analyses of the psychological condition were run to investigate whether predictors of situational trust were dependent on the environmental context (danger cues), perceived threat, and anxiety. We tested whether the dispositional trust measures predicted ratings of anxiety and threat, at each level of danger cue (testing H4a), followed by a test of whether the dispositional measures remained predictive of trust with anxiety and threat statistically controlled (testing H4b).

### Manipulation Checks

Analyses were run to check that the analysis mode and danger cue manipulations affected participants’ perceptions of the scenarios as intended. Participants rated the extent to which each scenario required a psychological judgment on a 1–5 scale. As expected, mean rating across all 24 scenarios was higher in the psychological condition (*M* = 3.24, *SD* = 0.86) than in the physics-based condition (*M* = 2.81, *SD* = 0.91), *t*(116) = 2.68, *p* < 0.01.

Table 2 shows mean anxiety and threat ratings at each level of Danger Cues. Each rating mean was averaged across the eight scenarios for each level (see Table 1). One-way, repeated-measures ANOVAs showed significant main effects of Danger Cues for threat rating, *F*(1.97,226.53) = 1287.21, *p* < 0.001, *η*^2^*_p_* = 0.918, and for anxiety rating, *F*(1.42,163.09) = 1287.21, *p* < 0.001, *η*^2^*_p_* = 0.551 (In these and subsequent ANOVAs, Box’s correction was applied to df’s when the sphericity assumption was violated). As the environment appeared more dangerous, ratings of threat and anxiety increased, as expected.

**Table 2:** Mean ratings for threat and anxiety at three levels of danger cues.

	Danger cues		
	Low	Medium	High
Threat rating	1.55 (0.39)	2.61 (0.45)	3.54 (0.47)
Anxiety rating	1.59 (0.55)	2.12 (0.83)	2.63 (1.11)

We also ran a series of Bonferroni-corrected *t*-tests to test whether participants allocated to the analysis mode conditions differed on any of the RoTA, PAS, NARS, and HIT scales. No significant differences were found. There were also no significant differences in variability in scale scores across conditions, tested with a series of *F* tests. Thus, the two participant groups appeared to be similarly composed in regard to the dispositional individual difference variables.

### Effects of Manipulations on Trust

The dependent variables for the analysis were means for the two rating scales of (1) confidence in the robot’s analysis and (2) likelihood of acting on the robot’s recommendation. Means were calculated for each cell of the design (see Table 1). Data were then analyzed with a 2 × 3 × 2 × 2 (Analysis Mode × Danger Cues × Robot Decision × Rating Scale) mixed-model ANOVA run using the SPSS Version 27 GLM procedure. Analysis Mode was a between-subjects factor, contrasting the two groups given physics-based or psychological transparency information. The remaining factors were within-subjects factors, contrasting, respectively, three levels of visible danger cues, the robot’s determination of threat or safety, and the two rating scales used.

The analysis showed main effects of Rating Scale, *F*(1,114) = 26.21, *p* < 0.001, *η*^2^*_p_* = 0.187, and Danger Cues, *F*(1.76,200.73) = 6.43, *p* < 0.01, *η*^2^*_p_* = 0.053. The Danger Cues main effect was modified by two significant interactions: Danger Cues × Robot Decision, *F*(1.78,202.47) = 231.82, *p* < 0.001, *η*^2^*_p_* = 0.670, and Analysis Mode × Danger Cues × Robot Decision, *F*(1.78,202.47) = 3.43, *p* < 0.05, *η*^2^*_p_* = 0.029. No other main effects or interactions attained significance.

**Figure 3:**
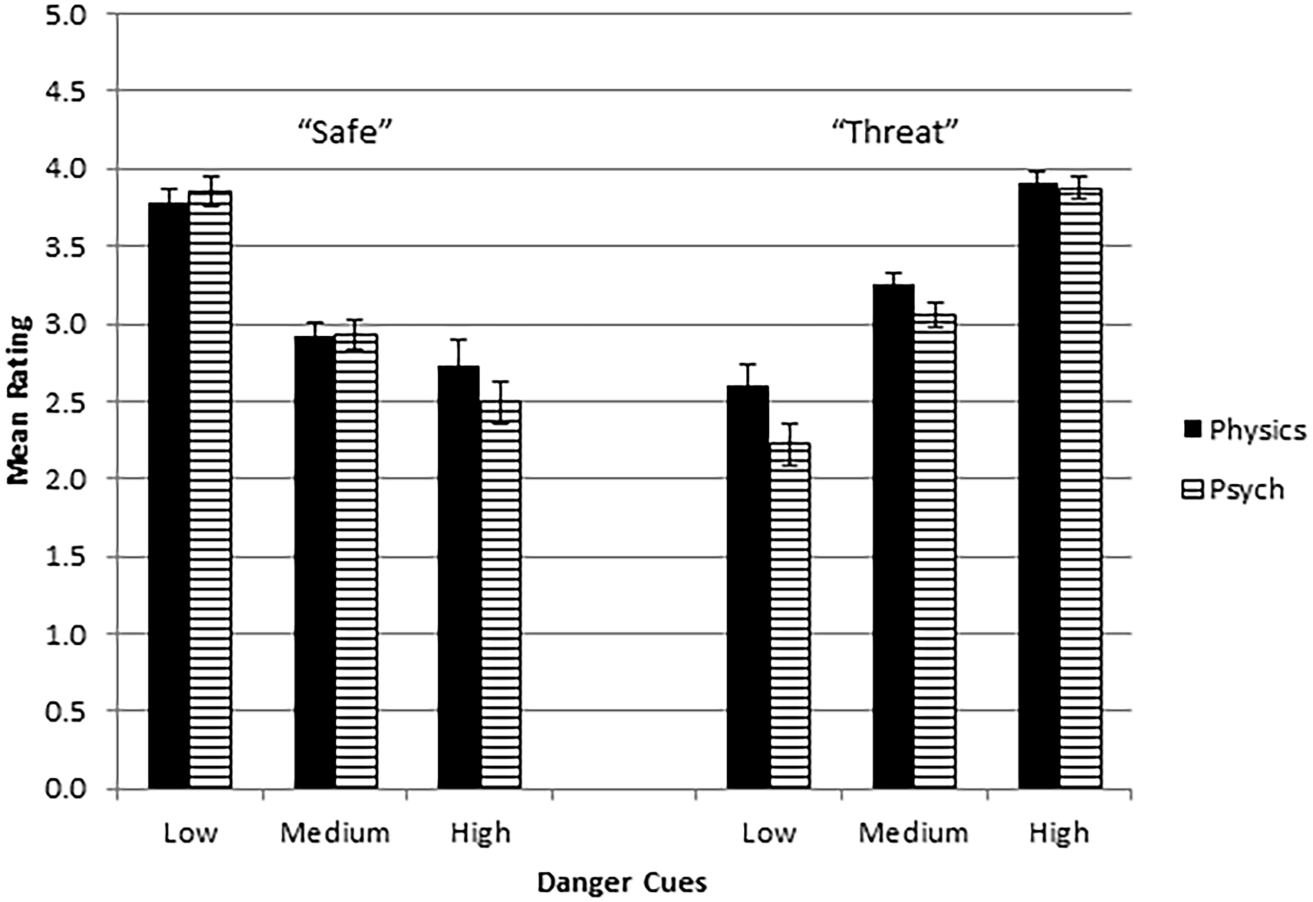
Estimated marginal means for trust rating as a function of level of danger cues, robot decision (“Safe” or “Threat”), and mode of analysis (Physics = physics-based, Psych = psychological). Error bars are SEs.

The main effect of Rating Scale was associated with higher ratings for confidence in the robot (*M* = 3.51, SE = 0.04) than for acting on its recommendations (*M* = 3.44, SE = 0.04). These values exceed the rating scale midpoint of 3. One-sample *t*-tests confirmed that mean ratings were significantly higher than 3.0 for confidence [*t*(117) = 11.38, *p* < 0.001, *d* = 1.05] and for acting on recommendations [*t*(117) = 10.37, *p* < 0.001, *d* = 0.95]. However, there were no significant interactions between the rating scale used and the other independent factors, implying that both scales measured a similar trust construct. The effects of the remaining independent variables are shown in Figure 3. The dependent variable is the GLM-estimated marginal mean, in effect the average of the two rating scales. The strongest effect was the crossover interaction between Danger Cues and Robot Decision. Trust ratings were higher when the robot’s decision matched the visible context, i.e., when the robot determined safety in a low-danger cue scene, or threat in a high-danger context. Similarly, mismatch produced lower trust. Ratings were generally similar in both physics-based and psychological transparency groups. The weak but significant three-way interaction primarily reflects higher trust when the robot used physics-based rather than psychological analysis to decide a low danger-cue scene was in fact threatening.

### Correlations Between Dispositional and Situational Trust Scales

#### Intercorrelations of Dispositional Scales

We analyzed the intercorrelations of dispositional scales related to trust to test whether they converged on a single, underlying general trust factor, or whether they assessed distinct personal qualities. Table 3 shows correlations between the dispositional scales for trust and attitudes toward robots available from the HIT, PAS, NARS, and RoTA scales. Scales showed substantial divergence, suggesting that they cannot be reduced to a single underlying trust dimension. The strongest cross-scale correlations were found between the HIT and NARS. Intentions to rely on robots, measured by the HIT, were lower in participants with negative attitudes, measured by the NARS, especially those with negative attitudes toward the social influence of robots.

**Table 3:** Intercorrelations of dispositional measures.

	1	2	3	4	5	6	7	8	9	10	11
1. PAS: high expectations											
2. PAS: all-or-none thinking	0.216^*^										
3. HIT	0.235^*^	−0.021									
4. RoTA-Phys: psychological judgment	0.110	0.108	0.076								
5. RoTA-Phys: confidence	0.149	0.040	0.265^**^	0.061							
6. RoTA-Phys: action recommen dations	0.072	−0.001	0.298^**^	0.056	0.813^**^						
7. RoTA-Psych: psychological judgment	−0.016	0.106	0.115	0.296^**^	0.223^*^	0.212^*^					
8. RoTA-Psych: confidence	0.261^**^	0.038	0.322^**^	0.251^**^	0.692^**^	0.605^**^	0.067				
9. RoTA-Psych: action recommen dations	0.180	0.032	0.311^**^	0.350^**^	0.542^**^	0.649^**^	0.058	0.869^**^			
10. NARS: interaction with robots	−0.062	0.146	−0.414^**^	0.189^*^	−0.167	−0.142	−0.096	−0.175	−0.140		
11. NARS: social influence of robots	−0.232^*^	0.043	−0.574^**^	−0.029	−0.165	−0.084	−0.011	−0.217^*^	−0.142	0.575^**^	
12. NARS: emotional interactions with robots	−0.158	0.065	−0.486^**^	−0.038	−0.115	−0.125	−0.014	−0.313^**^	−0.309^**^	0.620^**^	0.633^**^

* *p* < 0.05;

** *p* < 0.01.

#### Correlates of Situational Trust

Table 4 shows dispositional correlates of two measures of situational trust. The first is the mean of the participants’ ratings of confidence in robot analysis and likelihood of acting on its recommendations, averaged across all 24 scenarios. The use of the mean rating is justified because (1) the ANOVA showed no interactive effects on trust between Rating Scale and other factors and (2) the analysis and action recommendation means were highly correlated (*r* = 0.94). The second situational trust measure was the Schaefer (2016) TPS-HRI, providing a global evaluation across all scenarios. For both trust measures, Table 4 shows correlations for the whole sample and for each of the two analysis-mode groups separately. The “*z*” column provides the test for significance of the difference of the correlations for the two groups, performed using Fisher’s *r* to *z* transformation (McNemar, 1969). The table includes the dispositional measures of attitudes toward robots as well as the mean situational ratings of the scenarios.

**Table 4:** Correlates of two situational trust measures, overall and by analysis mode.

	Trust rating				TPI-HRI			
	All	Physics	Psychol.	*Z*	All	Physics	Psychol.	*z*
HRI	0.502^**^	0.492^**^	0.521^**^	0.21				
PAS: high expectations	−0.072	−0.199	0.056	0.77	−0.042	−0.077	−0.015	0.32
PAS: all-or-none thinking	−0.271^**^	−0.409^**^	−0.144	1.53	−0.086	−0.112	−0.062	0.27
HIT	0.257^**^	0.069	0.440^**^	2.13^*^	0.308^**^	0.244	0.376^**^	0.77
RoTA-Phys: psychological judgment	−0.128	−0.279^*^	0.007	1.55	−0.247^**^	−0.377^**^	−0.106	1.54
RoTA-Phys: confidence	0.286^**^	0.112	0.448^**^	1.96^*^	0.314^**^	0.234	0.398^**^	0.97
RoTA-Phys: action recommendations	0.299^**^	0.074	0.488^**^	2.43^*^	0.254^**^	0.120	0.391^**^	1.55
RoTA-Psych: psychological judgment	0.120	−0.052	0.296^*^	1.89	0.023	−0.035	0.093	0.068
RoTA-Psych: confidence	0.200^*^	−0.100	0.422^**^	2.91^**^	0.222^*^	0.024	0.407^**^	2.16^*^
RoTA-Psych: action recommendations	0.174	−0.159	0.432^**^	3.29^**^	0.127	−0.078	0.333^**^	2.25^*^
NARS: interaction with robots	−0.288^**^	−0.209	−0.376^**^	0.97	−0.307^**^	−0.348^**^	−0.256	0.59
NARS: social influence of robots	−0.087	−0.027	−0.172	0.78	−0.127	−0.107	−0.146	0.21
NARS: emotional interactions with robots	−0.094	0.074	−0.244	0.93	−0.208^*^	−0.139	−0.274^*^	0.15
Scenario threat rating	0.141	0.237	0.042	1.06	0.095	0.167	0.006	0.86
Scenario anxiety rating	−0.033	−0.132	0.046	0.53	−0.042	−0.045	−0.037	0.04
Psychological judgment rating	0.282^**^	0.089	0.504^**^	2.46^*^	0.096	0.037	0.155	0.63

* *p* < 0.05;

** *p* < 0.01.

Physics = Physics-based analysis mode, Psychol. = Psychological analysis mode, Z tests significance of difference of correlations in the two analysis modes.

In the whole sample, the HIT, the NARS negative attitude toward interactions subscale, and the majority of the RoTA subscales were consistent predictors of both situational trust measures. However, Table 4 also shows that predictors of situational trust varied across the two groups. The most consistent group differences were obtained with the RoTA-Psych scale. For both situational trust measures, the two RoTA-Psych measures were significantly correlated with trust only in the psychological transparency condition. The significant *z* statistics confirm that correlations differed across the two groups. The RoTA-Physics scale, surprisingly, showed a similar pattern of results, but the *z*s were significantly only for the trust rating measure, not for the TPS-HRI. The HIT was also more strongly correlated with situational trust in the psychological condition, with the *z* reaching significance for the trust rating measure only. The PAS high expectations scale was significantly negatively correlated with trust rating in the physics-based condition, but the *z* for the comparison with the psychological conditions was nonsignificant. The NARS scales tended to be negatively associated with trust, but there was no clear variation in correlation strengths across the two groups. The only situational rating to correlate with trust ratings was perceptions of the robot as making psychological judgments, which was more strongly correlated with trust in the psychological compared to the physics-based condition, as anticipated.

#### Regression Analyses

We used a regression approach to investigate predictors of the averaged situational trust rating. We investigated moderator effects of the robot’s analysis mode, by testing whether the regression of the trust criterion onto the individual-difference predictors differed in the two analysis mode conditions. Three regression models were tested. Model 1 tested for linear predictors of trust, and Models 2 and 3 added product terms to test for interaction between dispositional variables and analysis mode, i.e., moderator effects. Linear predictors were centered to avoid collinearity with interaction terms. Each model included at the first step a dummy variable for experimental condition (−1 = physics-based; 1 = psychological). The *R*^2^ of 0.003 at Step 1 was small and nonsignificant. Subsequent steps added linear and interaction terms to test contributions of predictor sets on a hierarchical basis. Model 1 was an initial test of the joint contribution of the full set of dispositional variables to predicting trust. To keep the model tractable, Model 2 utilized a reduced set of the most relevant predictors and tested the contribution of interaction terms computed as the product of experimental condition and the dispositional variable (H3a, H3b). Model 3 focused on the situational ratings of psychological judgment, threat and anxiety, and their linear and condition-dependent associations with trust (H3c).

Summary statistics for the regression models are given in Table 5. For Model 1, the dispositional predictors added around 25% to the variance explained, with scales from the PAS, HIT, and NARS making significant individual contributions, consistent with the correlational analysis. However, the change in *R*^2^ (Δ*R*^2^) likely reflects some chance associations due to the number of predictors. Model 2 thus included only those dispositional scales significantly associated with trust in one or other of the two experimental conditions, as specified in Table 4, i.e., PAS: all-or-none thinking, HIT, and NARS: interaction with robots. In Model 1, there was collinearity between ROTA scales, so Model 2 included only the two most theoretically relevant RoTA-Psych scales as predictors: Psychological Judgment and Confidence. Interaction terms were computed for each of the five selected dispositional predictors as the product of the centered linear term and the dummy variable for condition. The linear terms added 18.2% to the variance explained at Step 2, and the interaction terms a further 11.7% at Step 3. In the final equation at Step 3, the linear PAS and NARS terms were significant, and the interactions of condition and the two RoTA-Psych scales also made significant individual contributions. That is, the PAS and NARS scales predicted trust irrespective of the robot’s mode of analysis, but the relationship between RoTA-Psych and trust varied across the two conditions. The analysis of Model 3 showed significant contributions from both the linear situational ratings (12.3%) and the interaction terms (7.9%). The final equation at Step 3 showed significant contributions from both the linear and interactive terms for psychological judgment rating, but threat and anxiety appeared to be unrelated to trust.

**Table 5:** Regression statistics for three models for prediction of situational trust.

Model	Step 2				Step 3				
Model 1: full set of dispositional predictors	Linear terms		-						Individual predictors (at last step)
	*R*	df	Δ*R*^2^	df					
	0.519^**^	13, 104	0.267^**^	12, 104					PAS: all-or-none thinking (*β* = −0.22^*^) HIT (*β* = 0.24^*^) NARS: interaction with robots (*β* = −0.25^*^)
Model 2: reduced set of predictors with interactions	Linear terms				Interaction terms				
	*R*	df	Δ*R*^2^	df	*R*	df	Δ*R*^2^	df	
	0.430^**^	6, 111	0.182^**^	5, 111	0.550^**^	11, 106	0.117^**^	5, 106	PAS: all-or-none thinking (*β* = −0.22^*^) NARS: interaction with robots (*β* = −0.22^*^) Condition × RoTA-Psych: psychological (*β* = 0.19^*^) Condition × RoTA-Psych: confidence (*β* = 0.20^*^)
Model 3: scenario ratings with interaction	Linear terms				Interaction terms				
	*R*	df	Δ*R*^2^	df	*R*	df	Δ*R*^2^	df	
	0.355^**^	4, 113	0.123^**^	3, 113	0.453^**^	7, 110	0.079^*^	3, 110	Psychological rating (*β* = 0.32^**^) Condition × Psychological rating (*β* = 0.24^**^)

* *p* < 0.05

** *p* < 0.01.

**Figure 4:**
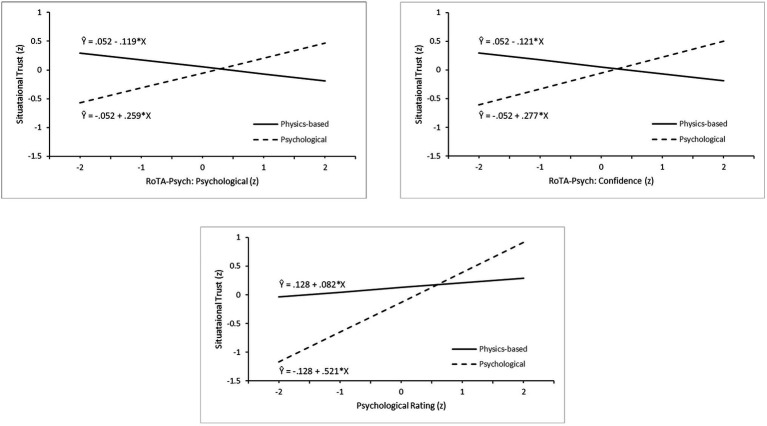
Regression plots for three interactive effects of robot analysis type and individual difference factors: Robot Threat Assessment (RoTA)-Psych psychological judgment scale (upper left), RoTA-Psych confidence scale (upper right), and scenario rating of robot psychological judgment (lower center).

Figure 4 illustrates the significant interactions identified in the analyses of Models 2 and 3. Each graph shows regressions of situational trust on the relevant predictor in the two experimental conditions, based on the standardized Step 3 regression equation. The two RoTA-Psych interactions show a similar pattern. In the physics-based condition, there was a negative relationship between trust and both psychological judgment and confidence in the robot’s psychological evaluations. In the psychological analysis condition, both RoTA-Psych variables show a stronger positive association with trust. Both believing that the security robots made psychological judgments and having confidence in those judgments appear to predispose the person to trust the robot in the scenarios, consistent with H3b. The Model 3 interaction shows that believing the robot was making a situational psychological judgment was only weakly positively correlated with trust in the physics-based experimental conditions, but the positive relationship was stronger in the psychological analysis condition, consistent with H3c.

### Intercorrelations of Trust, Situational Threat, and Anxiety: Psychological Condition

#### Dispositional Correlates of Threat and Anxiety

For these analyses, mean ratings of threat and anxiety were calculated separately for each level of danger cues, averaging across the eight scenarios at each level. Table 6 shows correlations between dispositional measures and threat and anxiety ratings. The RoTA was the strongest predictor of perceived threat, especially with high visible danger. Two of the NARS subscales were quite consistently associated with higher anxiety, but the correlations did not vary much across different danger-cue levels. Higher PAS subscale scores tended to be associated with lower anxiety, but the HIT was not predictive of either threat or anxiety.

**Table 6:** Correlations between dispositional measures and threat and anxiety ratings at three levels of danger cues, in psychological condition.

	Threat			Anxiety		
Danger cues	Low	Medium	High	Low	Medium	High
PAS: high expectations	−0.085	−0.205	0.082	−0.262^*^	−0.134	−0.075
PAS: all-or-none thinking	−0.076	−0.140	−0.075	−0.123	−0.301^*^	−0.290^*^
HIT	−0.067	−0.118	−0.031	−0.185	−0.150	−0.154
RoTA-Phys: confidence	−0.070	0.096	0.288^*^	0.117	0.040	0.042
RoTA-Phys: action recommendations	0.068	0.206	0.341^**^	0.061	0.042	0.062
RoTA-Psych: confidence	0.060	0.165	0.340^**^	0.036	0.081	0.169
RoTA-Psych: action recommendations	0.143	0.305^*^	0.385^**^	0.003	0.096	0.154
NARS: interaction with robots	−0.203	−0.048	−0.143	0.178	0.179	0.175
NARS: social influence of robots	0.058	0.299^*^	0.123	0.303^*^	0.349^**^	0.325^*^
NARS: emotional interactions with robots	−0.021	0.149	−0.040	0.331^*^	0.270^*^	0.228

* *p* < 0.05;

** *p* < 0.01.

### Correlates of Situational Trust by Danger Cue Level

In the psychological analysis mode condition, mean trust ratings were calculated separately for each level of danger cues, averaging ratings of confidence in robot analysis, and likelihood of acting on its recommendations across the eight scenarios for each level of danger cue. Table 7 shows correlations between the dispositional trust measures and mean trust rating at each level of danger cue. Generally, there was little variation in correlation magnitude across the levels. The strongest correlates of trust—HIT, RoTA, and NARS negative attitude toward interactions—were significantly correlated with trust at all three levels. Two of the NARS subscales were significantly correlated with trust only in the medium danger-cue condition. The table also provides partial correlations for the dispositional trust—situational trust correlations with mean threat rating and mean anxiety rating controlled. The partial correlations were generally similar in magnitude to the uncorrected correlations, suggesting that the validity of the dispositional measures as correlates of trust cannot be directly attributed to individual differences in trust and/or anxiety.

**Table 7:** Correlations between selected predictors and mean trust rating at each level of danger cues, in psychological condition.

	Danger cues					
	Low		Medium		High	
	R	Partial	r	Partial	r	Partial
PAS: high expectations	0.002	−0.045	0.079	0.093	0.099	0.061
PAS: all-or-none thinking	−0.073	−0.129	−0.155	−0.152	−0.173	−0.174
HIT	0.315^*^	0.335^*^	0.503^*^	0.517^**^	0.360^**^	0.481^**^
RoTA-Phys: confidence	0.349^**^	0.367^**^	0.444^**^	0.441^**^	0.396^**^	0.291^*^
RoTA-Phys: action recommendations	0.346^**^	0.443^**^	0.482^**^	0.484^**^	0.493^**^	0.383^**^
RoTA-Psych: confidence	0.266^*^	0.346^**^	0.435^**^	0.433^**^	0.441^**^	0.314^*^
RoTA-Psych: action recommendations	0.289^*^	0.427^**^	0.440^**^	0.447^**^	0.476^**^	0.331^*^
NARS: interaction with robots	−0.247	−0.444^**^	−0.422^**^	−0.435^**^	−0.344^**^	−0.335^*^
NARS: social influence of robots	−0.099	−0.091	−0.305^**^	−0.350^**^	−0.054	−0.166
NARS: emotional interactions with robots	−0.185	−0.253	−0.243^*^	−0.263^*^	−0.225	−0.258

* *p* < 0.05 and

** *p* < 0.01.

## Discussion

In this study, we investigated factors associated with trust in an advanced robot operating in a security role. Consistent with previous reviews (Hancock et al., 2011; Hoff and Bashir, 2015; Schaefer et al., 2016), we found that trust reflected multiple factors. In terms of model of Hancock et al. (2011), these included human factors (individual differences), robot factors (robot decision, mode of analysis), and an environmental factor (contextual threat). The current study confirmed that multiple individual difference factors may shape trust in the robot, in combination with robot and environmental factors.

The remainder of this section discuss the tests of the hypotheses in more depth. We found moderately high levels of trust in both physics-based and psychological conditions, together with sensitivity to contextual danger (see Section “Influences on Trust”). The young adult sample appeared to be generally quite rational in the assignment of trust, regardless of the robot’s mode of analysis. Scales that correlated with trust included NARS, PAS, and HIT, as hypothesized (see Section “Individual Differences in Trust”). The robot’s mode of analysis—physics-based or psychological—was more significant as a moderator of individual difference effects than as a direct influence on trust. In the psychological condition, we found that RoTA scales for perceiving security robots as making psychological judgments and confidence in those judgments predicted situational trust. These associations are consistent with activation of a “teammate” mental model enhancing trust when the robot is required to make human-like appraisals such as identifying negative emotions and aggressive intent. We also found individual differences in threat appraisal and anxiety, but these responses did not seem to directly drive variation in trust. Study findings have practical implications for optimizing trust in security settings (see Section “Applications to Selection and System Design”). Operator selection can include assessments on dispositional trust measures to identify individuals who allocate trust on a rational basis, taking into account both robot and contextual characteristics. Individual difference assessment can also guide personalization of training and delivery of transparency information. Study limitations including use of a novice sample, divergence of trust measures, and limited robot autonomy may be addressed in future research (see Section “Limitations”).

### Influences on Trust

Mean trust ratings of around 3.5 fell above the midpoint of the 1–5 scale, implying substantial but not perfect trust. Meta-analyses have shown that system reliability and performance is a major driver of trust (Hancock et al., 2011; Schaefer et al., 2016). On most trials, the robot’s evaluation was consistent with the threat context, which may have built confidence in its judgments. As hypothesized (H2), trust was highest, around four on the scale, when the visible level of environmental danger matched the robot’s judgment. Trust levels fell to about 2.5 when there was a mismatch between danger cues and robot decision. This value is a little below the scale midpoint, but greater than the lower anchor value of 1, implying that participants did not entirely dismiss the robot’s analysis. Thus, participants’ evaluations of the scenarios appeared rational in that they integrated both the robot’s decision and the environmental context, broadly consistent with the Bayesian perspective (Heuer, 1999).

We hypothesized that trust would reflect greater confidence in a robot utilizing physics-based analysis, relative to a “psychologizing” robot (H1). Contrary to the hypothesis, there was no significant main effect of analysis mode on either confidence in the robot’s judgment or likelihood of acting on its recommendation. At least in the security context, young adults do not seem to have any specific difficulty in trusting a robot making psychological evaluations from sensor data. Only when the robot judged there to be a threat in a low threat context scene was trust in the physics-based condition higher than in the psychology-based condition. Trust scores for this condition were based on perceptions of an unthreatening scene depicting office buildings with seven people standing near to a bus stop. In the physics-based condition, the robot reported threat based on analysis of data from an X-ray camera indicating a weapon and multiple unknown objects. In the psychological condition, threat was inferred from voice analysis indicating disorganized and agitated speech (inaudible to participant). Trust was relatively low in both conditions, but greater trust in the physics-based condition might reflect either greater belief in the robot’s ability to detect an imperceptible threat, or idiosyncratic features of the two versions of the scenario.

### Individual Differences in Trust

Previous work on trust in robots and autonomous systems suggests that situational trust may be shaped by the person’s stable mental model of robot functioning (Phillips et al., 2011; Ososky et al., 2014). Our previous work suggests that there are individual differences in the extent to which people are disposed to view robots as advanced tools vs. human-like teammates (Matthews et al., 2019). We assessed multiple dimensions of dispositional individual differences in trust, validated in previous research (Nomura et al., 2006; Merritt et al., 2015; Lyons and Guznov, 2018; Matthews et al., 2019). There was considerable divergence between the trust scales, implying a need to distinguish different aspects of the construct. Similar to Lyons and Guznov (2018), we found only weak associations between the PAS and HIT, suggesting that attitudes to automation diverge from those to robots. The HIT was quite substantially negatively correlated with all three NARS subscales, although the HIT was designed to assess trust as defined in the Mayer et al. (1995) model rather than negative emotion. At the dispositional level, trust in a robot partner may in part be shaped by emotional reactions. The HIT also correlated more strongly than did the PAS with the RoTA scales, suggesting that attitudes toward robot teaming generalize to the security context.

#### Linear Associations Between Dispositional Measures and Situational Trust

Correlates of situational trust were generally as anticipated. Associations were similar for the post-task Schaefer (2016) TPS-HRI and the rating-based trust measures, providing convergent validation. The HIT and RoTA were positively associated with trust, implying that high scorers on these scales encode positive features of robots in the mental model. Conversely, PAS and NARS subscales that represent negative attitudes were associated with lower trust. Data were generally consistent with previous studies linking higher trust in robots to higher HIT scores (Lyons and Guznov, 2018), to lower PAS all-or-none thinking scores (Merritt et al., 2015), and to lower NARS scores (Schaefer, 2013).

In the regression models, the PAS all-or-none thinking and NARS interaction with robots scales appeared to be the most robust predictors. The role of all-or-none thinking in undermining trust is consistent with view of Merritt et al. (2015) that this trait is damaging when the machine commits errors. However, contrasting predictions could be made about the impact of all-or-none thinking as systems become increasingly intelligent and autonomous. Security and military settings may require the robot to make decisions based on complex and incomplete information, so that errors are probable. In this case, humans expecting perfection in the machine are likely to lose trust rapidly. Alternatively, as robots are perceived as increasingly humanlike, human operators may become more forgiving of their errors, similar to how people are more tolerant of errors in humans than in conventional automation (Dzindolet et al., 2002; Merritt et al., 2015). In the present data, PAS all-or-none thinking was significantly correlated with lower trust in the physics-based but not the psychological condition, which suggests that participants were more likely to be perfectionistic when a tool rather than a teammate mental model was activated. However, the regression analysis failed to confirm a significant moderator effect of condition, so further work on this issue is required.

The NARS interaction with robots scale captures concerns about working with robots and the idea of a robot exercising judgment. The scale may have predicted lower trust here because the simulation was configured to portray the cognitive abilities of the robot in making inferences from sensor data. The additional NARS scales that assess discomfort with social and emotional robots might become more predictive if the robot was more emotionally expressive. For example, high scorers on these scales might reject expressions of reassurance from the robot that it was supporting the human’s mission goals (Panganiban et al., 2020).

Dispositional correlates of situational trust ratings were more numerous in the psychological than in the physics-based condition, perhaps reflecting the greater complexity of mental models for teammates relative to tools. Factors that related to judgment in the psychological condition included those relating to robots that act as teammates without being overtly human-like. For example, the HIT includes items referring to being comfortable with the robot taking responsibility and taking action without being monitored by the human. Such attitudes may be more strongly primed when the robot is analyzing sensor data for psychological attributes. Similarly, both physics-based and psychological RoTA scales, both of which refer to robots making complex evaluations, were associated with trust only in the psychological condition. The strongest correlate of trust in the physics-based condition, PAS all-or-nothing thinking, may be associated with a mental model for “dumb” tools being presumed to work perfectly.

#### Moderator Effects of Mode of Robot Analysis

We hypothesized (H3) that predictors of situational trust in the simulated environment would depend on the robot’s analysis mode. It was assumed that the mental model activated would depend on both the insights into robot functioning conveyed by the transparency messages and the person’s predispositions to consider the robot as a tool or a teammate. We considered that the PAS, HIT, and RoTA-Phys scales would assess attitudes to teaming encoded in the “advanced tool” mental model, and so would be more predictive of trust in the physics-based condition, relative to the psychological condition (H3a). The PAS all-or-none thinking scale was significantly negatively correlated with trust only in the physics-based condition, as expected. However, contrary to expectation, the HIT and RoTA-Phys scales were significantly correlated with greater trust only in the psychological condition. The regression analyses did not find significant moderator effects of condition for the PAS or HIT scales associated with trust in the bivariate analyses. Thus, these scales may be more effective in picking up general attitudes toward robots than utilization of the advanced tool mental model, although the PAS data showed a trend in the expected direction.

We also anticipated that the NARS and RoTA-Psych scales, which assess attitudes toward robots behaving in human-like ways, would predict trust only when the robot was in psychological analysis mode (H3b). Consistent with the hypothesis, the regression analysis showed significant moderator effects for two RoTA-Psych scales. In the psychological experimental condition only, trust was higher when the person was disposed to perceive the robot as making psychological judgments and to have confidence in “psychological” robots. Positive features of the mental model for security robots encourage trust in situational contexts in which the robot makes human-like threat assessments but remain latent when the robot functions as an advanced but conventional tool. By contrast, seeing security robots as “psychological” robot inspired mistrust in the physics-based condition, because the teammate model was incongruent with the robot’s mode of analysis. As previously discussed, the lack of congruence between the mental model and the robot’s functioning tends to lower trust (Ososky et al., 2014; Perelman et al., 2017).

The NARS scales tended correlate with lower trust, but they were not specifically associated with lower trust in the psychological condition, and the regression analysis failed to show any moderator effect for the NARS interaction with robots scale. The scale appears to represent a dislike of human-like robots that generalizes across contexts.

The participant’s rating of the extent to which the robot was making psychological judgments provides a measure of the extent to which a human-like teammate mental model was activated in the scenarios. As expected, ratings were higher in the psychological compared to the physics-based condition. In addition, supporting H3c, the rating was more strongly correlated with situational trust in the psychological than in the physics-based condition. The moderator effect for the situational rating, confirmed in the regression analysis, was similar to that for the dispositional rating of robots as psychological, assessed by the RoTA. Perceiving the robot as functioning in a human-like way enhanced trust in the appropriate context. However, in the absence of feedback, the human’s trust was never put to the test: the perception–trust link might be weaker if the robot’s judgments of humans were demonstrably erroneous.

#### Individual Differences in Emotional Response to Scenarios

The final issue addressed was the role of emotion in trust, focusing on the psychological condition. As explored in research of Nomura et al. (2006, 2008), some people react to robots with dislike and anxiety, factors that may undermine trust even if the robot performs competently. We hypothesized (H4a) that the NARS and RoTA-Psych scales would correlate with threat appraisal and anxiety ratings (in opposite directions), especially in the high threat-context condition, in which anxiety about the robot might combine with anxiety about the visible environment. This hypothesis was partially supported. The NARS correlated more consistently with anxiety than threat, whereas the RoTA showed significant negative associations with threat but not anxiety. As expected, the NARS primarily picks up emotion, whereas the RoTA is related to cognitive appraisal. Correlations between the RoTA and threat appraisal tended to increase with visible threat, but anxiety correlates of two NARS subscales, for social influence and emotional interactions, were fairly consistent across threat-context conditions. Both scales were associated with subjective reactions to interacting with the robot, but in different ways.

We computed partial correlations to test whether the NARS and RoTA remained correlated with trust, when anxiety and threat were statistically controlled. In fact, contrary to Hb4, correlations with trust were not much affected by controlling for these variables, implying that these individual differences in trust cannot be directly attributed to emotional response. Furthermore, the trust correlates of both NARS and RoTA were fairly consistent across different levels of threat context. Taken together, the current results elaborate on the constructs measured by the two instruments. The NARS interaction and RoTA subscales primarily assess beliefs about robot partners that are activated when the robot makes human-like judgments and influence trust but not emotion. The RoTA may encode additional beliefs about threat evaluation due to its contexualized nature. The NARS social influence and emotional interaction scales represent affective responses that may not be directly linked to underlying beliefs in robot attributes such as reliability, integrity, and benevolence (Mayer et al., 1995) that underpin trust.

### Applications to Selection and System Design

The novel human factor challenges of teaming with intelligent and autonomous machines raise concerns about trust optimization (Lyons and Havig, 2014; Shively et al., 2017; de Visser et al., 2018). Guidelines developed on the basis of research on conventional automation and robots may not transfer to robots behaving like human teammates. The present study provides some reassurance. Overall, trust levels were similar irrespective of the robot’s mode of analysis. Participants appeared to factor in environmental threat cues and the robot’s decision similarly for physics-based and psychological analyses.

However, analysis of individual differences suggested some more subtle applied concerns. Trust in the psychological condition appeared to be associated with a wider range of dispositional influences than was trust in the physics-based condition, suggesting possible biases in trust associated with preexisting beliefs about robots. The present study did not investigate how malleable these attitudes are; their influence may wane as the person becomes more familiar with the robot’s operations in a particular environment. If attitudes are persistent, the measures used here may have potential for personnel selection. It may be preferable to select operators with moderate scores on the HIT, NARS, and RoTA to filter out individuals with strong tendencies toward over- or under-trust of the robot. Alternatively, training may be personalized according to the person’s preexisting preferences, emphasizing robot competencies or error vulnerability according to the direction of bias.

In the present study, transparency was manipulated solely to prime one or other mental model. In the practical setting, more sophisticated forms of transparency than simple text messages are likely to be utilized (e.g., Chen et al., 2018). Nevertheless, the study indicates that transparency information can be tailored to individual difference factors. Transparency generally supports trust optimization (Lyons, 2013; Selkowitz et al., 2015), but the present data suggest that providing cues to the robot’s human-like attributes may backfire in some cases. For example, high scorers on the HIT and RoTA may be disposed to view the robot as a person-like teammate; emphasizing its human-like functions through transparency may lead to overestimation of its capabilities. For these individuals, transparency that highlights its fallibility as a computational machine may counter tendencies toward over-trust. Operators with low opinions of robots as teammates, and perhaps also high scorers on the NARS, may benefit from different forms of transparency. Information highlighting its computational competence may both increase trust directly, and limit activation of the negative “teammate” mental model. Another possible strategy for those averse to robots is to design transparency that highlights the benevolence and integrity of the robot, which may over time reshape the mental model adaptively.

### Limitations

The study has the limitations typical of investigating novice operators in a simulated environment. It is unclear whether results would generalize to samples of domain experts such as police officers or warfighters teaming with a robot in a real setting. However, current findings suggest the value of following up the current findings in a field setting. Some of the present individual difference measures have been validated in expert populations. For example, Lyons and Guznov (2018) utilized the PAS and HIT in a study of fighter pilots, and Bartneck et al. (2007) investigated the NARS in robot owners. More data are needed, especially from samples of security experts (cf., Gallimore et al., 2019). There are similar questions about the extent to which the simulated scenarios elicited the reactions that people would have to real security robots. If participants perceived the simulation as a video game, they may have made attributions about robot functioning that would not generalize to real life.

The limited convergence between alternate dispositional trust measures is a further challenge for research in this area. Trust is commonly considered as a unitary construct, and meta-analyses of influences on trust have treated it as such (Hancock et al., 2011; Schaefer et al., 2016). However, personal characteristics that influence trust appear to be more differentiated. We observed some convergence between the HIT and lower NARS scores, but associations between the PAS and RoTA and other dispositional measures were of moderate magnitude at best. Two factors may lead to differentiation of antecedents to trust. First, people may encode beliefs in the mental model that go beyond the trustworthiness of machines. Even at the tool level, people may differentiate attributes such as typical utility of the tool, utility across a range of contexts, susceptibility to damage, requirements for tool user skill, etc. Mental models for human and human-like teammates are even more complex. Thus, no single scale is likely to capture all the attributes of the mental model that shape trust. Second, the mental model may be context-dependent. The RoTA, although modestly correlated with HIT and NARS scales, may tend to dissociate from other measures because its items explicitly refer to the security context. Beliefs about security robots may differ from those about robots utilized in other contexts such as industry and healthcare (Savela et al., 2018). Thus, there is a need for additional psychometric research to identify the major generalized and context-specific dimensions, as well as qualitative studies for additional perspectives on variation in mental models (e.g., Lyons et al., 2019).

Another limitation is that there was no objective standard for trust calibration and optimization. Participants perceived occasional discrepancies between the visible environment and the robot’s threat evaluation, but they were not given direct information or feedback on the robot’s performance, i.e., its hit and false-positive rates. Thus, while the study identifies dispositional factors associated with potential for bias, we could not define the optimal level of trust. Future research might adopt an explicitly Bayesian approach, which would require that participants are aware of the base rate for threat and the robot’s performance levels. On the other hand, the base rate for threat may be objectively unknown in some field settings. Furthermore, visible danger cues are imperfectly diagnostic of true threat probabilities, and accurate threat analysis requires attention to cues to specific threats, such as improvised explosives, as well as to the more general environmental context (Zimmerman et al., 2014). Thus, the uncertainly common in realistic security contexts remains a challenge in optimizing trust in a robot partner.

The robot was also restricted in its autonomy and capacity for team-work. While participants were instructed that it used AI in making its threat determinations, it did not exhibit team-work behaviors such as choosing task goals on its own initiative or determining needs to provide back-up to the human. The simulation method employed here allows more complex scenarios to be modeled that would support investigation of trust in a robot exhibiting more autonomy. We found that participants rated confidence in the robot more highly than they did willingness to follow its action recommendations, irrespective of threat context and mode of analysis. A more complex simulation could secure behavioral measures of operator’s willingness to allow the robot to operate autonomously. Future work could also use a multiphase design to investigate changes in trust on a pre–post-basis, where the participant is given an initial preview of robot appearance and/or functionality, as recommended by Schaefer (2016). Such a design could also address trust repair following a robot error (e.g., de Visser et al., 2018).

## Conclusion

The present study confirms that trust in an intelligent robot performing security operations is appropriately sensitive to the robot’s behavior and contextual cues to threat. Transparency about the basis for the robot’s threat evaluations had only minor effects on trust. However, individual differences in trust varied according to the robot’s mode of threat analysis. When the robot performed human-like analyses of target persons’ motivations and intents, multiple dispositional factors associated with attitudes to robot teammates predicted trust. These correlates of trust may reflect attributes of a mental model for robots as teammates. They may signal biases in attitudes to robot partners that may lead to over- or under-trust. Strategies for optimal calibration of trust in robot teammates include using trust scales in personnel selection, personalizing training to counter bias, and personalizing transparency information during operations.

## Data Availability Statement

The datasets presented in this article are not readily available because it is subject to approval from United States Air Force. Requests to access the datasets should be directed to gmatthews@ist.ucf.edu.

## Ethics Statement

The studies involving human participants were reviewed and approved by University of Central Florida Institutional Review Board. The patients/participants provided their written informed consent to participate in this study.

## Author Contributions

All authors listed have made a substantial, direct, and intellectual contribution to the work and approved it for publication.

## Conflict of Interest

The authors declare that the research was conducted in the absence of any commercial or financial relationships that could be construed as a potential conflict of interest.

## Publisher’s Note

All claims expressed in this article are solely those of the authors and do not necessarily represent those of their affiliated organizations, or those of the publisher, the editors and the reviewers. Any product that may be evaluated in this article, or claim that may be made by its manufacturer, is not guaranteed or endorsed by the publisher.
